# CONTRACT Study - CONservative TReatment of Appendicitis in Children (feasibility): study protocol for a randomised controlled Trial

**DOI:** 10.1186/s13063-018-2520-z

**Published:** 2018-03-02

**Authors:** Natalie Hutchings, Wendy Wood, Isabel Reading, Erin Walker, Jane M. Blazeby, William van’t Hoff, Bridget Young, Esther M. Crawley, Simon Eaton, Maria Chorozoglou, Frances C. Sherratt, Lucy Beasant, Harriet Corbett, Michael P. Stanton, Simon Grist, Elizabeth Dixon, Nigel J. Hall

**Affiliations:** 1 Southampton Clinical Trials Unit, Faculty of Medicine, University of Southampton, Southampton, UK; 2 National Institute of Health Research (NIHR), Research Design Service South Central, University of Southampton, Southampton, UK; 3 Primary Care and Population Sciences, Faculty of Medicine, University of Southampton, Southampton, UK; 4 Great Ormond Street Hospital for Children NHS Foundation Trust, London, UK; 5 Centre for Surgical Research, Population Health Sciences, Bristol Medical School, University of Bristol, Bristol, UK; 6 Institute of Psychology, Health & Society, University of Liverpool, Liverpool, UK; 7 Centre for Child and Adolescent Health, School of Social and Community Medicine, University of Bristol, Bristol, UK; 8 UCL Great Ormond Street Institute of Child Health, London, UK; 9 Southampton Health Technology Assessment Centre, Faculty of Medicine, University of Southampton, Southampton, UK; 10 Department of Paediatric Surgery, Alder Hey Children’s NHS Foundation Trust, East Prescott Road, Liverpool, L14 5AB UK; 11 Department of Paediatric Surgery and Urology, Southampton Children’s Hospital, University Hospital Southampton NHS Foundation Trust, Southampton, UK; 12 Patient and Public Involvement Representative, Southampton, UK; 13 University Surgery Unit, Faculty of Medicine, University of Southampton, Mailpoint 816, Tremona Road, Southampton, SO16 6YD UK

**Keywords:** Appendicitis, Non-operative treatment, Paediatric surgery, Appendicectomy, Feasibility

## Abstract

**Background:**

Currently, the routine treatment for acute appendicitis in the United Kingdom is an appendicectomy. However, there is increasing scientific interest and research into non-operative treatment of appendicitis in adults and children. While a number of studies have investigated non-operative treatment of appendicitis in adults, this research cannot be applied to the paediatric population. Ultimately, we aim to perform a UK-based multicentre randomised controlled trial (RCT) to test the clinical and cost effectiveness of non-operative treatment of acute uncomplicated appendicitis in children, as compared with appendicectomy. First, we will undertake a feasibility study to assess the feasibility of performing such a trial.

**Methods/design:**

The study involves a feasibility RCT with a nested qualitative research to optimise recruitment as well as a health economic substudy. Children (aged 4–15 years inclusive) diagnosed with acute uncomplicated appendicitis that would normally be treated with an appendicectomy are eligible for the RCT. Exclusion criteria include clinical/radiological suspicion of perforated appendicitis, appendix mass or previous non-operative treatment of appendicitis. Participants will be randomised into one of two arms. Participants in the intervention arm are treated with antibiotics and regular clinical assessment to ensure clinical improvement. Participants in the control arm will receive appendicectomy. Randomisation will be minimised by age, sex, duration of symptoms and centre. Children and families who are approached for the RCT will be invited to participate in the embedded qualitative substudy, which includes recording of recruitment consultants and subsequent interviews with participants and non-participants and their families and recruiters. Analyses of these will inform interventions to optimise recruitment. The main study outcomes include recruitment rate (primary outcome), identification of strategies to optimise recruitment, performance of trial treatment pathways, clinical outcomes and safety of non-operative treatment. We have involved children, young people and parents in study design and delivery.

**Discussion:**

In this study we will explore the feasibility of performing a full efficacy RCT comparing non-operative treatment with appendicectomy in children with acute uncomplicated appendicitis. Factors determining success of the present study include recruitment rate, safety of non-operative treatment and adequate interest in the future RCT. Ultimately this feasibility study will form the foundation of the main RCT and reinforce its design.

**Trial registration:**

ISRCTN15830435. Registered on 8 February 2017.

**Electronic supplementary material:**

The online version of this article (10.1186/s13063-018-2520-z) contains supplementary material, which is available to authorized users.

## Background

Acute appendicitis is the most common surgical emergency in children [1]. The lifetime risk of developing appendicitis is 7–8%, and the most common age for developing appendicitis is in the early teens. Appendicectomy is considered the gold standard treatment for acute appendicitis by most surgeons, but many parents and patients find the prospect of the need for emergency surgery frightening and one they are keen to avoid if an alternative is available [2]. Preliminary work we have already undertaken with children and families confirms a high level of interest in non-operative treatment, and indeed a preference for non-operative treatment so long as clinical outcomes are comparable.

Although appendicectomy is considered a simple procedure, it requires a general anaesthetic and an abdominal operation with its associated risks. The complication rate of appendicectomy (including wound infection, intra-abdominal abscess and adhesional small bowel obstruction) is up to 25% [3], with a need for hospital readmission in 4–5% of cases [4, 5]. A contemporary estimation of these risks is available from the National Appendicectomy Audit, a nationwide audit of outcomes of appendicectomy for acute appendicitis in 19 specialist paediatric surgery centres in the United Kingdom [6]. Over a 2-month period, 242 appendicectomies for acute appendicitis were performed. The negative (histologically normal) appendicectomy rate was 10.3%, and the 30-day adverse event (AE) rate (a composite of readmission, re-intervention, pelvic collection and wound infection) was 15.3%. The economic burden to the healthcare system of paediatric appendicitis in England is in excess of £21 million per year and requires significant resource use, including need for out-of-hours surgery (45% of all paediatric appendicectomies were performed between 1800 and 0800 in the National Appendicectomy Audit).

An alternative approach to treating acute appendicitis in children would be treatment with antibiotics and without an appendicectomy. Whilst there is growing scientific interest in the use of non-operative treatment with antibiotics owing to its potential benefits over surgery and existing data to support its safety, the relative efficacy of this approach compared with appendicectomy is not yet known [7]. By undergoing a non-operative approach to treatment of their appendicitis, patients may avoid the mental and physical stress and trauma of an operation as well as the associated complications. Non-operative treatment has the potential to reduce the quantity of resources used by the National Health Service (NHS). For example, by reducing the amount of theatre time, staff time and surgical resources used for the treatment of appendicitis, there could be significant savings for the NHS.

It has been known for some time that acute appendicitis can be treated successfully by antibiotics alone in the context of remote environments without surgical service capability [8]. However, the role of non-operative treatment as primary therapy has only recently come under consideration in developed healthcare systems, initially in adults [3, 9–15] and more recently in children [16–18]. Although studies in adults are sometimes extrapolated to children, to do so is problematic because there are key differences in appendicitis occurring in adults compared with in children. The presentation of appendicitis and the intra-abdominal inflammatory response are different in adults and children [19, 20] and may be more amenable to antibiotic treatment alone, and the psychosocial and economic impact of appendicitis in children affects the whole family rather than just the individual. Therefore, a paediatric randomised controlled trial (RCT) is necessary to compare both treatment options.

There has been just one pilot RCT, recently performed in Sweden, comparing non-operative treatment with antibiotics with appendicectomy in children with acute appendicitis [18]. Fifty children (aged 5–15 years) with acute non-perforated appendicitis were randomised to antibiotics (*n* = 24) or appendicectomy (*n* = 26). All children in the surgery group had histopathologically confirmed acute appendicitis, and none experienced a significant surgical complication. In the antibiotic group, 2 of 24 underwent appendicectomy within the time of primary antibiotic treatment, and 1 further child required appendicectomy for histologically proven, recurrent acute appendicitis 9 months later. Of the eligible participants, the recruitment rate was 40%; the drop-out rate following treatment allocation was 2% (1 patient); and no patient was lost to follow-up by 1 year. This pilot study was not powered sufficiently to compare the efficacy of antibiotics versus surgery, but it was conducted to inform the design of an international, multicentre RCT which is currently recruiting in non-UK centres [21].

Our group recently performed a systematic review and meta-analysis comparing the efficacy of non-operative treatment and appendicectomy for uncomplicated appendicitis in children [7]. Whilst there were limitations related to a lack of RCTs, the existing data support a position of equipoise between these two treatment approaches. Neither our review nor any of the contributing studies [16–18, 22–24] identified any safety concerns regarding non-operative treatment.

In addition to outcomes of the acute illness, the development of recurrent appendicitis is an important consideration in children who receive non-operative treatment that is not applicable to children treated with appendicectomy. In adults [9–12, 25], the incidence of recurrence (within 1 year) is around 15%. A recent pilot study of non-operative treatment of appendicitis in children with 1 year of follow-up reported a recurrence rate of 5% [18], and our recent systematic review estimated an incidence of 14% [7]. This is the best current estimate in children.

Given the current uncertainty regarding the relative efficacy and cost-effectiveness of non-operative treatment compared with appendicectomy in children with uncomplicated acute appendicitis, a definitive RCT is necessary. Although RCTs are ongoing in other countries [26, 27], there are important differences in diagnostic techniques and healthcare delivery in the United Kingdom that mandate a UK-specific trial. These include a much lower reliance on diagnostic imaging for confirmation of appendicitis in the United Kingdom than in other countries, as well as a higher negative appendicectomy rate and a lower uptake of laparoscopic appendicectomy in the United Kingdom, all of which may influence relative efficacy of non-operative treatment compared with surgery [6, 28]. Prior to performing a large efficacy trial, we designed this feasibility study, which includes a feasibility RCT, to inform the design and conduct of a future RCT and establish whether a main trial is possible in the United Kingdom.

## Methods/design

### Study design

The CONTRACT (CONservative TReatment of Appendicitis in Children Trial - feasibility) study comprises the following elements:

1. A randomised controlled feasibility trial of children comparing a non-operative treatment pathway with appendicectomy. A standardised treatment pathway (Fig. 1) will be used in each arm of the study, beginning with broad-spectrum antibiotics from the point of enrolment. One arm will then undergo urgent appendicectomy, while the other will be treated non-operatively with continuation of broad-spectrum antibiotics. Both treatment pathways will include the same follow-up schedule.
2. A detailed programme of embedded qualitative and quantitative research to optimise recruitment to the feasibility RCT. It will also inform the design and conduct of any future RCT of non-operative treatment versus appendicectomy in the treatment of acute uncomplicated appendicitis in children.
3. A health economics (HE) feasibility study to allow the identification of key cost drivers and other parameters necessary to perform a full economic evaluation in our future RCT. This will include the design and piloting of data collection tools and adoption of a micro-costing approach. A full protocol of the HE substudy is described separately.
4. The development of a core outcome set (COS) for the non-operative treatment of children with uncomplicated acute appendicitis for use in the future RCT as well as in the wider research community. A full protocol for the COS is published elsewhere [29].
5. A patient and public involvement (PPI) work stream that reciprocally feeds into elements 1, 2 and 4 above. We have formed a study-specific advisory group (SSAG) made up of children who have had acute uncomplicated appendicitis, children who have not, and parents.

#### Randomised controlled feasibility trial

##### Population

The sample population will comprise children aged 4–15 years inclusive with a clinical diagnosis of acute appendicitis who would normally be treated with an appendicectomy as part of their standard care. Patients will be identified by the clinical team at the time of diagnosis, and their eligibility will be confirmed by the research team as soon as possible.

##### Inclusion criteria

- Children aged 4–15 years (> 3 and < 16 years)
- Clinical diagnosis, either with or without radiological assessment, of acute appendicitis which prior to study commencement would have been treated with appendicectomy
- Written informed parental consent, with child assent if appropriate

##### Exclusion criteria

- Clinical signs or radiological findings to suggest perforated appendicitis
- Presentation with appendix mass
- Previous episode of appendicitis or appendix mass treated non-operatively
- Major anaesthetic risk precluding allocation to the appendicectomy arm
- Known antibiotic allergy preventing allocation to non-operative treatment arm
- Antibiotic treatment started at referring institution (defined as two or more doses administered)
- Cystic fibrosis
- Positive pregnancy test
- Current treatment for malignancy

##### Randomisation

Eligible patients will be identified, approached and consented by the treating clinician. After written informed consent is obtained, a member of the trial team on-site will randomise the participant to one of two treatment groups in a 1:1 ratio via an independent web-based system (Trans European Network for Clinical Trials Services [TENALEA]). This online system allows complete pre-randomisation concealment of treatment allocation and provides instant assignment to either the appendicectomy or non-operative treatment group. Minimisation will be used to account for recruiting centre and ensure balance between the groups in factors that may affect diagnostic accuracy and outcome of treatment. The factors which are taken into account are (1) sex (male or female), (2) aged 4–8 or 9–15 years, (3) duration of symptoms (onset of pain to recruitment into study < 48 h or ≥ 48 h) and (4) recruiting centre.

##### Interventions

**Fig. 1:**
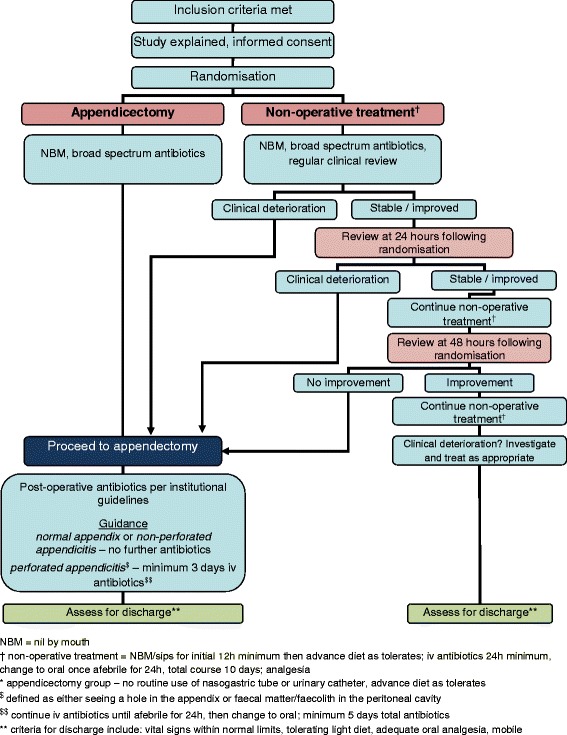
Clinical pathway for both treatment arms

##### Non-operative treatment group

The treatment pathway in the non-operative treatment group will comprise fluid resuscitation, a minimum of 24 h of broad-spectrum intravenous (IV) antibiotics (per local policies), a minimum of 12 h of nil by mouth (NBM) and regular clinical review to detect signs and symptoms of significant clinical deterioration, including but not limited to increasing fever, increasing tachycardia, and increasing tenderness. After the initial 12 h period of NBM, oral intake will be advanced as tolerated. Children successfully treated without an operation will be converted to oral antibiotics once they are afebrile for 24 h and tolerating oral intake (per local policies and after the minimum 24 h broad-spectrum IV antibiotics).

Clinical reviews will also be completed at approximately 24 h and 48 h post-randomisation. Any children who show signs of significant clinical deterioration by 24 h or at any point during the trial will undergo appendicectomy. Children who are considered stable or improving will continue with non-operative treatment. At 48 h, any children who have not shown clinical improvement will also undergo appendicectomy. The decision to continue non-operative treatment at these time points or to recommend discontinuation of non-operative treatment and appendicectomy will be made by the treating consultant and based on clinical judgement rather than on any specific features that are not evidence-based. All reasons for change in treatment will be recorded in detail.

Any children who receive an appendicectomy for an incomplete response to non-operative treatment will follow a standardised post-operative treatment regimen already in use at each institution and identical to that used in the appendicectomy arm. The reason for having an appendicectomy will be recorded.

##### Appendicectomy group

Children randomised to the appendicectomy arm will undergo either open or laparoscopic appendicectomy at the surgeon’s discretion, performed by a suitably experienced trainee (as per routine current practice) or a consultant. A peritoneal microbiology swab will be taken at the time the peritoneum is first opened or from the appendix, and from any peritoneal fluid sent for microbiological culture. The results of this swab will be recorded.

Patients will receive IV antibiotics from the time of randomisation and be treated post-operatively with IV antibiotics according to existing institutional protocols; however, the following recommended regimen is used to guide practice: children with acute uncomplicated appendicitis or a macroscopically normal appendix will receive no further antibiotics. Children with a perforated appendix (defined as a faecolith or faecal matter within the peritoneal cavity, or visualisation of a hole in the appendix) will continue to receive IV antibiotics for a minimum of 3 days and will receive a minimum total course of antibiotics of 5 days (IV and oral). It is not possible to standardise the duration of antibiotic therapy, owing to anticipated variation in intra-operative findings and in response to treatment. The types of antibiotics used will be identical to those used in the non-operative treatment arm within each centre. Any child failing to respond to these first-line antibiotics will be treated as is clinically appropriate with a longer course of antibiotics or a change in antibiotic therapy, with the choice of antibiotic determined by intra-operative swab or fluid culture.

Post-operatively, children with uncomplicated acute appendicitis or a normal appendix will not routinely have a nasogastric tube or a urinary catheter. They will receive oral intake as tolerated after surgery.

##### Discharge assessment

Criteria for discharge to home will be identical to those in both treatment groups and will be as follows: vital signs within normal limits for age, afebrile for ≥ 24 h, tolerating light diet orally, adequate oral pain relief and be mobile. Patients being treated non-operatively will receive a total course of 10 days of antibiotics following randomisation, unless decided otherwise by the clinician. If more than 10 days of oral antibiotics are administered, this will be recorded (including reason). Children who receive non-operative treatment will not routinely be offered interval appendicectomy, but they will be counselled about the risk of recurrence using best available data.

Once a decision to discharge the child has been made, a member of the clinical team who has not been involved directly in the child’s treatment will be asked to complete a discharge assessment. This assessor will not have prior knowledge of the randomisation or treatment received by the child. Upon completion of the discharge assessment, they will “guess” which treatment the child received. If the assessor should become unblinded during the assessment, this will also be recorded. Through this we hope to be able to determine the feasibility of a blinded discharge assessment in a future RCT.

##### Follow-up

**Fig. 2:**
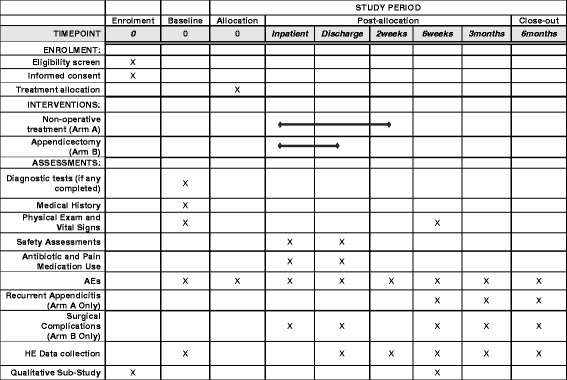
Standard Protocol Items: Recommendations for Interventional Trials (SPIRIT) checklist: patient schedule of procedures. *AE* Adverse event, *HE* Health economics

Follow-up appointments for all participants will take place at 6 weeks and at 3 and 6 months following discharge, either in the outpatient clinic or in the clinical research facility at each centre. If a face-to-face appointment is not possible, the 3- and 6-month follow-up can be completed over the phone. Data on resource use, time to return to daily activities and recurrent appendix-related problems (including unexplained abdominal pain and recurrence) will be collected prospectively to ensure high accuracy. The schedule of enrolment, intervention and follow-up is shown in Fig. 2.

##### Primary outcome

The primary outcome is assessment of whether it is feasible to conduct a multi-centre RCT testing the effectiveness and cost-effectiveness of a non-operative treatment pathway for the treatment of acute uncomplicated appendicitis in children. This will be evaluated as the proportion of eligible patients who are approached and recruited to the study over 12 months.

##### Secondary outcomes

The following secondary outcomes are centred predominately on the qualitative and COS substudies contributing to the development of a future RCT:

1. Willingness of parents, children and surgeons to take part in a randomised study comparing operative versus non-operative treatment and identify anticipated recruitment rate. This will be assessed from audio-recorded family-surgeon recruitment consultations; interviews with patients, parents, surgeons and nurses; surgeon surveys; and focus groups.
2. Identification of strategies to optimise surgeon-family communication using the above consultation and interview data.
3. Design of a future RCT from the perspectives of stakeholders at participating sites (e.g., children, parents, surgeons, nurses) informed by the consultation and interview data, surgeon surveys and focus groups.
4. Assessment of the equipoise and willingness of UK paediatric surgeons to participate in a future RCT through surgeon surveys and focus groups.
5. Clinical outcomes of trial treatment pathways, including the following:

- Overall success of initial non-operative treatment (measured as the number of patients randomised to non-operative treatment, discharged from hospital without appendicectomy)
- Complications of disease and treatment (measured during hospital stay and 6-month follow-up period)
- Rate of recurrent appendicitis during 6-month follow-up period

6. Performance of study procedures including retention of participants for the duration of the study, and feasibility of outcome recording and data collection systems.

##### Sample size calculations

The study will recruit participants from three centres for 12 months. Each centre treats 80–100 children per year with acute appendicitis, with an estimate that at least 130 will be eligible out of the 240–300 potential patients. Assuming that 40–50% will be recruited (i.e., 52–65 participants in feasibility RCT), we will be able to estimate a true 40% recruitment rate with a 95% CI of 31–49% and a true 50% recruitment rate with a 95% CI of 41–59%. A total of 52–65 participants in the feasibility RCT will be adequate to test treatment pathway procedures, data collection methods and loss to follow-up. For the embedded qualitative work related to recruitment, we will recruit until we reach data saturation, which we estimate will entail analysing approximately 40 recruitment consultations, 20–30 family interviews and 20–25 healthcare professional interviews.

##### Clinical trial data analysis

Data analysis will be performed by the study statistician, who will be blinded to treatment allocation by the use of coded data as per the statistical analysis plan. Because this is a feasibility study, all analyses will be treated as preliminary and exploratory and will be mainly descriptive. Feasibility outcomes (number of eligible patients, recruitment/retention rates, reasons for non-participation, success of blinding of the discharge assessor), treatment outcomes and complications will be presented by simple summary statistics with 95% CIs. Clinical outcome measures will be compared between treatment groups in an exploratory analysis, and variability estimates will be used to inform the sample size for a future definitive trial. The study will be reported in accordance with the Consolidated Standards of Reporting Trials (CONSORT) 2010 statement.

##### Trial oversight and safety monitoring

A study management group (SMG) will be responsible for overseeing the day-to-day management of the trial. A trial steering committee (TSC) and data and safety monitoring committee (DSMC) will also share independent oversight of the study. The DSMC will review the trial and its data from a safety and ethical perspective and will make recommendations regarding the continuation of the trial to the TSC, who will make the ultimate decision. The roles and responsibilities of each committee are detailed in a separate charter. The SMG will provide feedback to the SSAG and vice versa.

Any patient who does not complete the non-operative treatment pathway within the trial (i.e., deteriorates or does not improve) and undergoes appendicectomy will be reported to the trial manager (TM) within 48 h of appendicectomy. The TM will inform the TSC chairperson and convey the clinical data relating to this patient. The TSC chair will hold responsibility for determining whether to ask the DSMC to meet and review the data from that patient. The DSMC will subsequently advise the TSC on their findings, including an assessment of whether it is acceptable to continue to recruit patients.

#### Qualitative substudy

The embedded qualitative substudy comprises audio recordings of recruitment consultations between patients, their families and recruiters (paediatric surgeons and research nurses), as well as follow-up interviews with patients, their families and recruiters about their experiences of recruitment and the trial. Focus groups will also be conducted with paediatric surgeons at non-study sites about their views of the trial. When patients are approached about the study, they will be asked for verbal consent to audio-record the discussion. Seeking written consent for the audio recording at this point would distract from the focus of the consultation; therefore, we will ask patients at the end of the consultation for written consent to keep the recording and use it for analysis. After discharge, a trained qualitative researcher will contact and invite patients and their families to be interviewed either in their homes or by telephone. Recruiters will also be invited to be interviewed either in their place of work or by telephone. All consultations and interviews will be digitally audio-recorded and uploaded for transcription by a professional transcription service and pseudo-anonymised before analysis.

##### Analysis of recruitment consultations

Analyses of the recruitment consultations will use both the recordings and transcripts to document the interactions between recruiters and families, explore information provision and use of communication techniques, as well as intervention preferences and trial participation decisions. If analyses of the audio-recordings suggest that recruitment difficulties are potentially linked to communication during the recruitment consultation, this information will be fed back to the local principal investigators so that training of recruiters can be implemented immediately. The equipoise and views of healthcare professionals recruiting to the trial will also be assessed, as well as the key ways in which their views differ from non-participating surgeons.

The analyses will also draw upon content analytic methods to describe what was said by whom and how often in the audio recordings of recruitment sessions. Constant comparison methods will also inform identification of common or divergent themes, particularly focusing on the impact of statements by the recruiter on parent responses and views. This will focus on key sections of the transcripts, such as when randomisation is offered. The percentage of eligible patients recruited will be documented using site screening logs, noting any families who decline randomisation or do not accept the randomised allocation.

##### Analysis of interview and focus group data

The findings derived from the analysis of the recruitment consultations will be linked with qualitative data from the interviews where patients discuss the acceptability of trial methodology to determine the feasibility and acceptability of a full trial, and also with the recruiter interviews. Analysis of interview and focus group data will draw on the principles of the constant comparative method and thematic analysis. One member of the research team will lead a process of ‘cycling’ between the developing analysis and new data. Other members of the qualitative study team (including at least one surgeon) will develop and test the analysis by periodic discussion and independent analyses of a proportion of transcripts to compare coding and findings.

Initially, each transcript will be read several times by the lead analyst before development of open codes to describe each relevant unit of meaning, although coding will occur at multiple levels, from detailed descriptions of communication and experiences of the trial, to the general orientation of participants towards clinical research. Through comparison within and across the transcripts, the open codes will be developed into categories to reflect and test the developing analysis. The categories will be organised into a framework to code and index the transcripts using NVivo software (QSR International, Doncaster, Australia). The framework categories will continually be checked and modified to ensure an adequate ‘fit’ with the data, whilst also accounting for variation in the data and ‘deviant’ cases. A second member of the team will check the categories and the assignment of data to them. Our analytic approach will be informed by writings on quality in qualitative research [30].

### Patient and public involvement

We recognise that PPI is a crucial element of this study, and as such, we will form a SSAG made up of parents, children and young people, some of whom will have experience of treatment for acute uncomplicated appendicitis. This group will provide overarching consultation and collaboration functions for the programme of research, minus the HE substudy. The group will help devise patient and parent documentation (including but not limited to information sheets, consent forms and a recruitment video) and provide insight on the qualitative substudy interview schedule and COS development. They will also help with the dissemination of the results back to study participants via information sources accessed by children, young people and parents and through a variety of media.

## Discussion

### Progression to main trial

Through this initial study we aim to inform the design, conduct and feasibility of a future efficacy RCT whilst confirming for the first time the safety of non-operative treatment in UK paediatric surgical centres. The decision to progress to a future RCT will be based on a combination of recruitment rate achieved, safety of non-operative treatment and adequate surgeon interest. These issues will be discussed by the trial management and oversight groups and be reviewed by a new funding panel. Currently, we think that a future main RCT will be considered feasible if the following goals are met:

1. The lower boundary of the 95% CI of the recruitment rate is above 20%. Whilst it is likely there are adequate patients to complete a study in which the recruitment rate is less than 20%, this is interpreted as lack of patient interest in non-operative treatment or, potentially, concerns about the trial and associated treatment.
2. The DSMC does not stop the trial on safety grounds. If the DSMC chooses to stop the trial, the non-operative treatment pathway will have to be reconsidered before a future RCT is planned.
3. Adequate surgeons and centres can be identified that are required to achieve target recruitment. On the basis of the current sample size estimate, five to ten UK paediatric surgery centres are required to make a future RCT feasible.

### Specific ethical considerations

1. Participants will be randomised to a novel care pathway, which, although in use at a number of institutions worldwide, has not been rigorously tested to assess efficacy and safety in participating centres. Although existing literature proposes that the non-operative pathway is safe [7, 16, 24], patients and their families will be informed that the clinical outcomes are being investigated as part of the study. Clinical reviews have been incorporated into the treatment pathways to minimise risk and/or complications of unsuccessful treatment.
2. Although written informed consent will be given by the parent or guardian, the child will be given age-appropriate information about the study and may confirm their assent during the completion of the consent form if they wish to do so. Consent will be taken by a member of the surgical team who has experience recruiting children to research studies and has completed appropriate good clinical practice training. A copy of the study consent form is included with the study protocol (*see* Additional file 1).
3. Owing to the urgency associated with the treatment of appendicitis, the period for taking consent will be short to ensure that the research process does not impede upon the provision of safe and effective care, but it does allow sufficient time for patients and their family to make an informed decision about the trial.

Some patients/parents may be concerned that delay in appendicectomy may increase the rate of perforation and AEs. However, this is not borne out by the literature on large numbers of adult [31] and paediatric patients [31–35], and participants will be counselled accordingly.

Following treatment, children in the non-operative treatment group will theoretically continue to be at risk of recurrence of appendicitis. Whilst the risk of recurrence is low, the children and their families will be fully informed of this risk. We will seek permission from these families to hold their personal details in a secured registry and to contact them in the future to determine if they have had a recurrence.

## Trial status

This study protocol describes version 2, dated 10 April 2017. The study opened to recruitment on 1 March 2017 and will recruit patients for a period of 12 months until 28 February 2018 at three paediatric surgical teaching hospitals in England: Alder Hey Children’s Hospital, Liverpool; Southampton Children's Hospital, Southampton; and St. George’s Hospital, London.

## Additional files

Additional file 1: Parental consent form with optional patient assent. (DOC 136 kb)

Additional file 2: SPIRIT checklist. (DOC 123 kb)

## Abbreviations

AE: Adverse event
CONSORT: Consolidated Standards of Reporting Trials
CONTRACT: CONservative TReatment of Appendicitis in Children - randomised controlled Trial
COS: Core outcome set
DSMC: Data and safety monitoring committee
HE: Health economics
IV: Intravenous
NBM: Nil by mouth
NHS: National Health Service
NIHR: National Institute for Health Research
PPI: Patient and public involvement
RCT: Randomised controlled trial
SMG: Study management group
SPIRIT: Standard Protocol Items: Recommendations for Interventional Trials
SSAG: Study-specific advisory group
TENALEA: Trans European Network for Clinical Trials Services
TM: Trial manager
TSC: Trial steering committee
