# Supplementary material for: CONTRACT Study - CONservative TReatment of Appendicitis in Children (feasibility): study protocol for a randomised controlled Trial
Source: Trials. 2018 Mar 2;19:153. doi: 10.1186/s13063-018-2520-z (PMC5833142; doi:10.1186/s13063-018-2520-z)
Supplement: Supplementary file 1 — Parental consent form with optional patient assent. (DOC 136 kb) [file 13063_2018_2520_MOESM1_ESM.doc]

Centre Number: Participant Trial ID Number:

**CONSENT FORM**

**CONservative TReatment of Appendicitis in Children**

**– a randomised controlled Trial (Feasibility)**

**Please initial box**

1. I confirm that I have read the Information Sheet dated.................... (version............) for the
   above study. I have had the opportunity to consider the information, ask questions and I have
   had these answered satisfactorily.
2. I understand that my child’s participation is voluntary and that I am free to withdraw them at any

time without giving any reason and without my child’s medical care or legal rights being affected.

1. I understand that relevant sections of my child’s medical notes and data collected during
   the study, may be looked at by individuals from the NHS Trust treating my child, the

Southampton Clinical Trials Unit, University Hospital Southampton NHS Trust and Regulatory

Authorities, where it is relevant to my child taking part in this research. I give permission for

these individuals to have access to my child’s records. I understand that only those individuals

who need to see this information will have access and all information will remain confidential.

1. I understand that the information collected about my child will be used to inform our future

research, and may be shared anonymously with other researchers.

1. I agree to my child’s General Practitioner being informed of their participation in the study.
2. I consent to be contacted by the research team as is required for the study, and I understand

that mine and my child’s contact details (name, address, telephone number) will be stored

securely and will not be revealed to anyone outside of the study team.

1. I agree to my child taking part in the above study.
2. I understand that neither myself nor my child will benefit financially even if future research leads

to the development of new treatments or alterations of procedures

1. I give permission for my recorded consent discussion to be used in the study. I understand

that my name will not be used in this process and that any quotes used will remain anonymous.

(Optional)

1. I confirm that I am happy for my contact details to be passed to the research assistant and to

be contacted to discuss the communication study as explained in the Patient Information

Sheet. (Optional)

Name of Parent / Guardian Date Signature

Name of Person taking consent Date Signature

**Child Assent**

Child’s assent to participation is optional for children who have read either the Information Sheet for Teenagers (version.........date….....) or the Information Sheet for Young People (version.........date….....).

Name of child Date Signature

**Contact details for Communication Study:**

Email address:

Telephone numbers: (preferred) /

Please provide two phone numbers and an email address if possible

*Reminder for research team: When completed, 1 copy to patient/parents, 1 copy in patient’s medical notes, original in site trial file.*
